# Shift in Black Rhinoceros Diet in the Presence of Elephant: Evidence for Competition?

**DOI:** 10.1371/journal.pone.0069771

**Published:** 2013-07-17

**Authors:** Marietjie Landman, David S. Schoeman, Graham I. H. Kerley

**Affiliations:** 1 Centre for African Conservation Ecology, Department of Zoology, Nelson Mandela Metropolitan University, Port Elizabeth, South Africa; 2 Faculty of Science, Health, Education and Engineering, University of the Sunshine Coast, Maroochydore, Queensland, Australia; Bangor University, United Kingdom

## Abstract

In African large herbivore assemblages, megaherbivores dominate the biomass and utilise the greatest share of available resources. Consequently, they are considered a separate trophic guild that structures the food niches of coexisting large herbivores. However, there exists little empirical evidence on how food resources are shared within this guild, and none for direct competition for food between megaherbivores. Using the histological analysis of faeces, we explore this phenomenon for African elephant *Loxodonta africana* and black rhinoceros *Diceros bicornis* in the Addo Elephant National Park, South Africa, where the accumulated impacts of elephant have reduced browse availability. Despite being unable to generalise beyond our study sites, our observations support the predictions of competition theory (as opposed to optimality theory) by showing (1) a clear seasonal separation in resource use between these megaherbivores that increased as resource availability declined, and (2) rhinoceros changed their selectivity in the absence of elephant (using an adjacent site) by expanding and shifting their diet along the grass-browse continuum, and in relation to availability. Although black rhinoceros are generally considered strict browsers, the most significant shift in diet occurred as rhinoceros increased their preferences for grasses in the presence of elephant. We speculate that the lack of specialised grazing adaptations may increase foraging costs in rhinoceros, through reduced harvest- and handling-efficiencies of grasses. In the short-term, this may be off-set by an enhanced tolerance for low quality food and by seasonally mobilising fat reserves; however, the long-term fitness consequences require further study. Our data suggest that managing elephant at high densities may compromise the foraging opportunities of coexisting browsers. This may be particularly important in small, fenced areas and overlapping preferred habitats where impacts intensify.

## Introduction

Since Sinclair [1] suggested that large mammalian herbivores are primarily food-limited (as opposed to predator-limited – [2], [3]), the importance of competition and niche separation in structuring these species' assemblages is widely recognised [4], [5]. In African large herbivore assemblages, megaherbivores (i.e. species weighing>1000 kg as adults, including African elephant *Loxodonta africana* and black rhinoceros *Diceros bicornis*) typically dominate the biomass and utilise the greatest share of the available resources through their enhanced tolerance of lower-quality food [6]. Consequently, they are considered a separate trophic guild that plays a key role in the abundance and structure of mesoherbivore communities [7], [8], and hence ecosystem functioning (*sensu* [9]). These trophic interactions are particularly significant for elephant and are mediated mostly by powerful effects on vegetation structure and composition (reviewed in [10]). For some mesoherbivores, the impacts facilitate access to habitat and increase the availability and quality of food [6], [11]. As an example, the conversion of tall woodlands to shrub coppice improves access to nutrient-rich regrowth for browsers. However, where elephant are abundant, they may be considered keystone competitors (*sensu* [12]) that regulate resource utilisation in local communities, thus limiting large herbivore abundances [7], [8]. Nevertheless, despite the fact that megaherbivores (particularly elephant) dominate resources and structure the food niches of mesoherbivores, there exists little empirical evidence on how resources are shared within this trophic guild (e.g. [13]–[15]), and none for direct competition for food between megaherbivores. Understanding the role of these bottom-up controls is particularly important for this guild, which is relatively invulnerable to top-down processes (e.g. predation – except that imposed by humans, disease; [2], [6]).

Because ecologically similar species are unable to coexist indefinitely on the same resources, interspecific competition is expected to promote the use of different resources [16]–[18]. Amongst the megaherbivores, elephant and black rhinoceros coexist in diverse habitats (e.g. woodlands, grasslands, semi-deserts) and share similar foods owing to wide feeding tolerances for abundant items of varying structure (e.g. leaves, twigs, bark) and nutritional quality [6]. Nevertheless, elephant are mixed-feeders that utilise browse and green grass, depending on seasonal availability, while black rhinoceros are strict browsers that select mostly dwarf shrubs, succulents and forbs with their prehensile upper-lip [6], [10]. Thus, when seasonal resources are reduced (e.g. grasses and forbs during the dry season), the coexistence of these herbivores on woody browse is presumably facilitated by their enhanced tolerance of lower-quality food, provided that the quantity is not limiting. However, where elephant movements (seasonal and long-term dispersal) are constrained by fences, interspecific competition (exploitative and interference) may intensify as populations expand, effects on woody communities increase, and browse availability declines. In these cases, competition is expected to be asymmetric in favour of elephant, owing to their larger size (elephant:rhinoceros body mass ratio: ♀ 3∶1, ♂ 5∶1), which confers an advantage in terms of the costs of agonistic interactions [19], [20]. The competitive ability of elephant is further enhanced by their greater foraging capacity (e.g. felling trees to access branch tips or roots) and ability to achieve high rates of food intake (through simultaneous handling and chewing), across a wide vertical range (up to 8 m above-ground versus<2 m for rhinoceros); these advantages reflect specialised foraging adaptations such as the mobile trunk [6], [10].

Implicit in the theory that interspecific competition promotes the use of different resources (as opposed to complete exclusion), is the understanding that shifts in resource use may be correlated with the intensity of competition [16]–[18]. In particular, demonstrating such shifts in response to the presence of a potential competitor is considered to be direct evidence of competition [18]. In the succulent thickets of the Addo Elephant National Park, South Africa, elephant dominate large herbivore biomass and population densities have exceeded (2–8 fold) recommended levels for 50 years [21]. As a consequence, elephant effects on the woody community are dramatic, and significant declines in species richness, density and biomass have been recorded (reviewed in [21], [22]). We expected these long-term impacts and high elephant densities to limit food availability for coexisting browsers, thus increasing the potential for competition. Our study tested this for black rhinoceros by (1) describing the seasonal diet and dietary preferences of coexisting elephant and rhinoceros to determine how resources are shared within this guild, (2) assessing the degree of diet separation in relation to the seasonal availability of resources, and (3) contrasting the diet and preferences of rhinoceros in the presence and absence of elephant (using adjacent sites). We predicted that if competition is important in shaping the food niche of rhinoceros then (1) diet separation should increase towards the dry season (late autumn-winter) when seasonal resources are reduced and both diets converge on browse [17], and (2) through competitive release, rhinoceros should broaden their diet and shift their preferences (by including more preferred foods and/or excluding non-preferred items) in the absence of elephant [16], [18]. It is possible that the predicted change in diet may simply reflect differences in the availability of resources between sites (i.e. a site-effect). To account for this potential constraint, we further tested our results against the predictions of optimality theory in which (1) diet breadth is inversely correlated with the availability of resources (i.e. rhinoceros should maintain a restricted diet in the absence of elephant), and (2) preferences do not respond to a change in availability, unless selectivity changes [23]. Finally, we measured the nutritional costs of the predicted shift in resource use with faecal quality descriptors and discuss our results in terms of the potential consequences for coexisting megaherbivores in small, enclosed areas.

## Methods

### Ethics statement

Because our research did not involve the capture, handling or disturbance of elephant or rhinoceros, neither our institute, nor South African National Parks required our research to pass through an ethics procedure. We nevertheless undertook our study with utmost consideration for the animals and their environment, collecting only samples of faecal matter for analysis. South African National Parks permitted us to conduct this study in the Addo Elephant National Park.

### Study site

The study was conducted in adjacent fenced sections of the Addo Elephant National Park (33°31'S, 25°45'E), South Africa. At the time of the study (2001–2003), 11 black rhinoceros and nearly 400 elephant coexisted in the Addo Main Camp section (AMC; 120 km^2^), while seven rhinoceros were located *c.* 1.5 km north in a 7 km^2^ area. No elephant were present at this site. The sites were generally similar except for the long-term (*c.* 50 years) browsing effects of elephant in AMC. Besides the megaherbivores, both sites supported a diverse mesobrowser community (5 spp.), dominated by kudu *Tragelaphus strepsiceros*. Rhinoceros in the Addo Elephant National Park are managed as a metapopulation with sub-populations elsewhere in the region.

The region is semi-arid with 260–530 mm rainfall annually, peaking in late-spring (November) and early-autumn (March). Nutrient-rich soils give rise to succulent thicket habitats [24], which covered *c*. 70–80% of the study sites. These thickets are typically evergreen, 2–4 m high, dense, thorny and dominated by the tree succulent *Portulacaria afra*. The remaining habitat at the sites comprised a mosaic of thicket, karoo and riverine types with grasslands derived from previous agricultural use. The vegetation is characterized by a high diversity of growth forms: drought-resistant succulents (e.g. *P. afra*), low trees (e.g. *Euclea undulata*, *Schotia afra, Sideroxylon inerme*) and spinescent woody shrubs (e.g. *Azima tetracantha, Capparis sepiaria, Carissa bispinosa, Gymnosporia* spp., *Searsia* spp.) contribute the bulk of plant biomass, while the understory hosts dwarf succulents, forbs, geophytes and perennial grasses. Couch grass *Cynodon dactylon* is seasonally abundant in grasslands and areas where intensive utilization by elephant has removed the canopy shrubs [22].

### Diet composition

We determined the diet of elephant and rhinoceros by identifying plant epidermal fragments in faeces [25]. Reference slides of the epidermal tissues of>350 potential food items at the sites were available for comparison. The technique is used extensively to contrast diets (e.g. [26], [27]) and its accuracies and biases are summarized in Holechek et al. [28]. Although faecal analysis may be biased toward less digestible food items in ruminants, these biases are likely to be reduced in megaherbivores with relatively poor digestion [6], [28]. Thus, we considered contrasts in fragment representation between herbivores and sites as valid indicators of dietary differences.

Fresh faecal samples were collected seasonally from November 2002-June 2003 (for elephant and rhinoceros in AMC) and August 2001-April 2002 (for rhinoceros, elephant absent). Four seasons were distinguished based on patterns of temperature, rainfall and frost: spring (September–November); summer (December-February); autumn (March-May); winter (June-August). Elephant faeces were collected opportunistically from family groups, while rhinoceros faeces were collected from latrines throughout the sites. Because the sites were located in close proximity (*c*. 1.5 km apart) and rainfall did not vary greatly between sample periods (i.e. between 387 and 321 mm during 2001/2 and 2002/3, respectively), we expected differences in rhinoceros diet to reflect a response to the effects of elephant, rather than sample period. Faeces were oven-dried and prepared following Landman et al. [29]. We identified 100 epidermal fragments to species level per faecal sample and treated each sample as an independent observation. In total, 41 elephant (10–11 samples per season) and 35 (elephant present; 8–9 samples per season) and 33 (elephant absent; 8–9 samples per season) rhinoceros faecal samples were analyzed. The diets were described as the frequency-of-occurrence of all the recorded plant species. Plant nomenclature follows the most recent list of southern African plants [30].

### Food availability

Relative food availability was estimated by measuring plant canopy cover (e.g. [27], [29]). Twenty 50 m line-transects were placed randomly and in proportion to the occurrence of habitat types at each site, during the wet (spring) and dry (late autumn-winter) season. Although most succulent thicket shrubs are evergreen, many grasses and forbs and some geophytes become dormant during the dry season [24], hence the need for the seasonal approach. We considered all food items encountered along transects as potentially available to elephant, but limited food availability for rhinoceros to items that occurred below their estimated maximum foraging height (175 cm; [31]). Only *c.* 19% of the browse measured in this way in AMC occurred beyond the reach of rhinoceros, and was therefore exclusively available to elephant.

### Diet quality

We estimated rhinoceros diet quality between sites by measuring faecal nitrogen (N_f_), phosphorous (P_f_) and crude fibre (NDF_f_) concentrations. Nitrogen and phosphorous availability is widely limiting to herbivore growth, reproduction and the maintenance of body condition (e.g. [32]). We randomly selected 15 faecal samples from each site and measured N_f_ using the Kjeldahl method [33], P_f_ using inductively coupled plasma spectrometry, and NDF_f_ according to the methods of Goering and Van Soest [34]. Sample analyses were conducted by the Grootfontein Agricultural Development Institute (N_f_) and KwaZulu-Natal Department of Agriculture (P_f_, NDF_f_), South Africa. Concentrations are expressed as percent dry matter.

### Data analysis

We generated accumulation curves (50 random iterations) of plant species recorded per faecal sample with which to assess the adequacy of sample sizes. Because none of the accumulation curves reached a stable plateau, the non-parametric Incidence-based Coverage Estimator [35] was used to estimate total dietary richness. Differences between observed and expected counts provided an estimate of the variation in dietary information at the upper limit of sampling effort.

Elephant and rhinoceros diets were contrasted seasonally using principal dietary items (PDI) and by grouping all plant species into broad growth form categories (i.e. grasses, woody shrubs, succulents, forbs, lianas, geophytes and epiphytes); we combined the seasonal data to contrast rhinoceros diets between sites. Our approach of using PDI was based on the observation that 64% (rhinoceros) and 74% (elephant) of the plant species utilized during the study contributed<1% each to the diets, presumably as many are incidentally browsed.

Foods consumed in the greatest quantities (abundances; [36]) and which collectively contributed most of the variation in dietary information were considered PDI. These were identified by ranking plant species in decreasing order of abundance, plotting their cumulative contribution to the diet, and scoring the slope of this curve relative to that at the origin (i.e. the contribution of the dominant item). PDI were considered to be those for which the slope of the cumulative curve was at least 10% of that at the origin: beyond this point, each plant species contributed relatively little to the diet. This is more objective than the approach of Petrides [36] in which an arbitrary cut-off based on the contribution of each species was used. We used non-metric Multidimensional Scaling (n-MDS) ordinations, based on Bray-Curtis resemblance matrices [37], [38], to visualise differences in the utilization of PDI across seasons and between sites. Each point on a biplot represents the data from one faecal sample. Data were square-root transformed to down-weight the influence of abundant items and the fit of each ordination was assessed with a *Stress* value; we corroborated ordinations with a *Stress*>0.20 with hierarchical agglomerative cluster analyses [37]. A non-parametric Analysis of Similarity (ANOSIM; 5000 Monte Carlo permutations) was used to test the null hypothesis of no difference in the utilization of PDI between groups. The *R* statistic ranges between zero and one, representing low and high discrimination between groups, respectively. *R* values were used as an index of the extent of dietary separation between elephant and rhinoceros for each season and trends (across seasons) were verified using conventional indices of resource overlap (e.g. [39]). Multivariate analyses were performed with *Primer* Version 6 [38].

Differences between the consumption and relative availability of food items (i.e. preferences for plant species or groups) were assessed by calculating 95% confidence intervals for the mean utilization of each item [40]. In principle, we considered food items to be preferred if utilization was greater than availability (i.e. subtracting percent availability from percent utilization resulted in a positive value) and the lower confidence limit was greater than zero (where use = availability); negative values indicated avoidance. Preferences were calculated by combining the relative availability and utilization data across seasons.

ANOVA procedures (Tukeys' test) were used to test differences in the use of growth forms across seasons and between sites. Where appropriate, percentage data were arcsine-transformed for normality and heteroscedasticity of variances.

## Results

### Food availability

We recorded 145 plant species, comprising mainly woody shrubs (37%), forbs (18%) and succulents (17%) along transects and quantified their relative availability for elephant and rhinoceros; sixty percent of the recorded species were shared between sites. Although food availability is expected to decline to a minimum during the dry season (particularly, grasses, forbs and some geophytes), we detected no difference in the relative abundance of growth forms between seasons for elephant (*F*_6,266_ = 0.66, *P* = 0.681) or rhinoceros (with a narrower foraging height range; *F*_6,266_ = 0.60, *P* = 0.728) in AMC. Food availability for rhinoceros varied between sites (*F*_6,553_ = 24.38, *P*<0.001): specifically, grasses were significantly more abundant in AMC (18.5% vs. 42.7%), while the reverse was true for woody shrubs (36.7% vs. 56.3%).

### Diet composition

In total, we identified 90 plant species in the diet of elephant and 92 (elephant present) and 87 (elephant absent) species in the diet of rhinoceros (Fig. S1). These species accounted for *c.* 87–95% of the estimated richness at the upper limit of sampling effort, confirming that the sample sizes used here were adequate to describe and compare the diets.

#### Diet separation between coexisting elephant and rhinoceros

**Figure 1:**
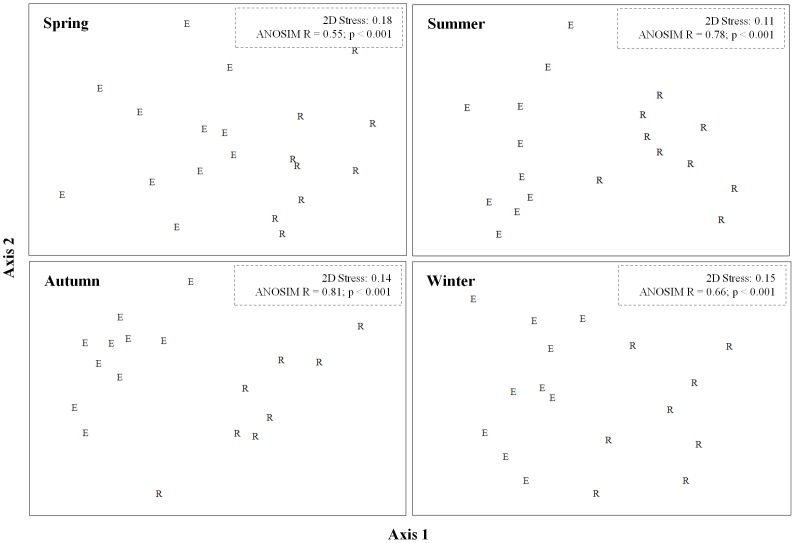
Non-metric Multidimensional Scaling ordinations of principal dietary items identified in the diet of elephant (E) and black rhinoceros (R) in the Addo Main Camp section. ANOSIM (Analysis of Similarity) *R* values indicate the degree of diet separation across seasons; values approaching unity indicate clear separation.

Only 18% (elephant) and 26% (rhinoceros) of the recorded plant species were utilised extensively, contributing 72–77% of the diets, and were thus considered PDI (Table S1). N-MDS ordinations showed a clear separation between elephant and rhinoceros in their use of PDI across seasons (Fig. 1), with a high degree of dissimilarity (53–63%), which was statistically significant (*P*<0.001) in each instance. Diet separation increased from spring (ANOSIM *R* = 0.55) through summer (ANOSIM *R* = 0.78) to autumn (ANOSIM *R* = 0.81; Table S1). This corresponded with a decline in the number of shared PDI: from 16 shared in spring to only 6 in autumn, comprising 11 and 5 woody shrubs, respectively.

**Figure 2:**
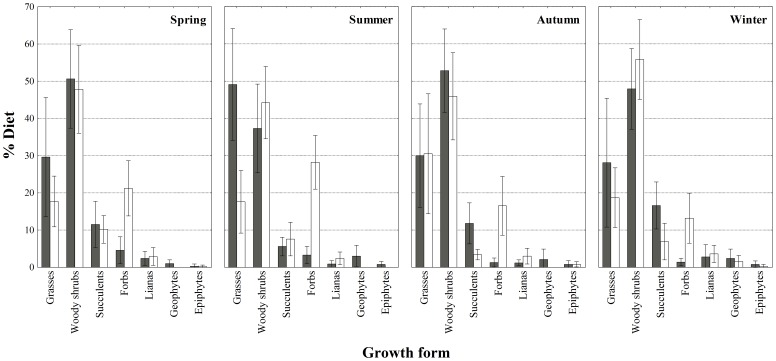
Seasonal diet, grouped into broad growth form categories (mean ± SD), of elephant (shaded bars) and black rhinoceros (clear bars) in the Addo Main Camp section.

**Figure 3:**
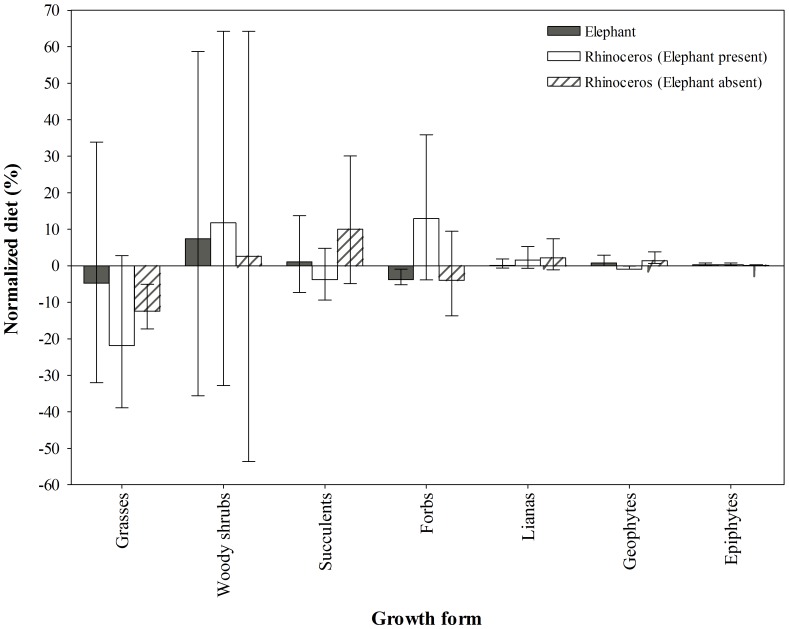
Normalized diet, grouped into broad growth form categories (mean ± 95% confidence interval), of elephant and black rhinoceros in the Addo Elephant National Park. Positive values (i.e. use>availability) with lower confidence limits greater than zero indicate preference; negative values (use<availability) indicate avoidance; at zero, use = availability.

Woody shrubs were the most diverse group identified (elephant: 40 spp.; rhinoceros: 42 spp.) and formed equal proportions of the bulk of the diets in all seasons (Fig. 2). As expected, the diets diverged most noticeably with respect to growth forms that may only be available ephemerally, specifically grasses and forbs (*F*_18,476_ = 3.88, *P*<0.001). Across seasons, rhinoceros utilised significantly more forbs, while elephant utilised more grasses during summer. Elephant also decreased their use of grasses and rhinoceros their use of forbs significantly from summer to winter (Fig. 2), possibly in response to a decline in the availability (or quality) of these groups. This provided circumstantial evidence that the pattern of increased diet separation coincided with a decline in food availability, and possibly the avoidance of competition. Elephant compensated for the decline in grass utilisation by increasing their use of succulents (Fig. 2), particularly *P. afra* (summer: 4.2%; winter: 15.1%). With the exception of epiphytes and geophytes, we observed no differences between the consumption and relative availability of growth forms (*P*>0.05; Fig. 3). Both herbivores preferred epiphytes (*P*<0.05), while only rhinoceros avoided geophytes (*P*<0.05).

Overall, approximately 69% (elephant) and 46% (rhinoceros) of the PDI were shared (11 spp.), comprising mostly woody shrubs (7 spp.), but also the dominant food item (*C. dactylon*) in both diets (Table S1). Forbs were PDI only for rhinoceros, but of these, only *Chascanum cuneifolium* was never recorded in elephant diet (and thus utilised exclusively by rhinoceros). We observed similar preferences for shared PDI with only *C. bispinosa* preferred by rhinoceros (*P*<0.05), but not elephant (*P*>0.05; Table S1).

#### Diet shift in rhinoceros

The n-MDS ordination showed a clear difference in rhinoceros diet between sites (60% dissimilarity; Fig. S2), which was statistically significant (ANOSIM *R* = 0.69, *P*<0.001). In line with the predictions of competition theory (as opposed to optimality theory), rhinoceros increased their diet breadth (estimated using PDI) by nearly 80% in the absence of elephant (paired t-test: *t*_(1)66_ = 17.40, *P*<0.001; Table S1). This was caused by an increase in the use of woody shrubs (11 spp., 73%), succulents (3 spp., 150%) and forbs (2 spp., 50%), while lianas (4 spp.) were only PDI where elephant were absent.

The proportion of growth forms that contributed the bulk of the diets varied significantly between sites (*F*_6,462_ = 38.78, *P*<0.001). In the absence of elephant, rhinoceros increased their use of woody shrubs (58.9% vs. 48.5%) and succulents (17.5% vs. 7.1%) and decreased their use of forbs (11.3% vs. 19.9%). Surprisingly, where elephant were present, grasses (mean: 20.8%, range: 8–63%) were particularly abundant (Fig. 2) and the diet was dominated (Table S1) by the short mat-forming grass *C. dactylon* (mean: 13.7%, range: 5–35%). These abundances were at least three times greater than those from the adjacent site (mean: 6.1%, range: 1–12%) without elephant. Although *C. dactylon* (5.2%) also featured as a PDI in the absence of elephant (Table S1), the tree succulent *Euphorbia triangularis* (5.9%) and the spinescent woody shrub *A. tetracantha* (5.2%) were equally dominant. Despite the seasonal decrease in grass consumption shown by elephant in AMC (Fig. 2), presumably due to a decline in availability, rhinoceros maintained high levels of use (only different from elephant in summer; *F*_18,476_ = 3.88, *P*<0.001) in all seasons (range: 17.6–30.5%). We thought that grass consumption may have been incidental and thus related to the utilisation of forbs and low-growing succulents. However, there was no correlation between the proportion of grass in the diet and that of these groups (*r_s_* = −0.26, *n* = 35, *P* = 0.138), suggesting selection for grasses. Relative to availability, rhinoceros decreased their preferences for grasses between sites (Fig. 3), such that these were avoided foods (*P*<0.05) where elephant were absent. Preferences for the remaining groups were similar between sites, with only geophytes showing a switch (*P*<0.05) from avoided (elephant present) to preferred (elephant absent).

Twenty PDI were shared between sites, mostly woody shrubs (13 spp.; Table S1). Few PDI were exclusively used at either site, despite these being present at both sites. We detected no difference in the preferences for PDI (Table S1) shared between sites (*χ* ^2^_2_ = 1.10, *P* = 0.577) or with elephant (*χ* ^2^_2_ = 1.50, *P* = 0.472).

### Diet quality

Despite the significant shift in rhinoceros diet between sites, N_f_ (elephant present: mean = 1.1%, SE = 0.1%; elephant absent: mean = 1.0%, SE = 0.1%) and NDF_f_ (elephant present: mean = 91.2%, SE = 1.3%; elephant absent: mean = 90.3%, SE = 1.0%) concentrations did not change (N_f_: *t*_(2)1,28_ = 1.11, *P* = 0.275; NDF_f_: *t*_(2)1,28_ = 0.54, *P* = 0.593). However, P_f_ levels were significantly lower (*t*_(2)1,28_ = −4.23, *P*<0.001) at sites where elephant were present (mean = 0.14%, SE = 0.01%), than at those where elephant were absent (mean = 0.20%, SE = 0.01%).

Because we detected no difference in N_f_ or NDF_f_ concentrations between sites, we hypothesised that grass utilisation played a positive role in the maintenance of constant diet quality. Results showed a significant positive relationship between the proportion of grass in the diet (varying from 1–47% between samples tested) and N_f_ levels (*R* ^2^ = 0.24, *F*_1,28_ = 8.93, *P* = 0.006; N_f_ = 0.92+(0.01 * % Grass), but no relationship with NDF_f_ (*R* ^2^ = 0.01, *F*_1,28_ = 0.13, *P* = 0.725) or P_f_ (*R* ^2^ = 0.03, *F*_1,28_ = 0.71, *P* = 0.408). Note that because the proportion of grass and browse in the diet is inversely related, the above relationships are similarly related for browse.

## Discussion

Despite extensive evidence of the effects of elephant on food resources in the Addo Elephant National Park [22], [41], [42] and elsewhere [6], [10], [43], few studies have investigated the consequences of this for other large herbivores. Surprisingly, where this information exists, the emphasis has been on demonstrating that elephant facilitate herbivore access to habitat and increase the availability and quality of food [6], [11]. This is despite clear evidence that elephant limit herbivore abundances across ecosystems through their ability to monopolise resources [3], [8]. Our study is the first to suggest direct competition for food with elephant, and by testing this for black rhinoceros, albeit at only one reserve, we provide insights into the potential role of competition in structuring the megaherbivore guild.

Although we are unable at present to generalise beyond AMC, our results comprise two lines of evidence that support the predictions of competition theory (as opposed to optimality theory; [16]–[18]). First, we show a clear separation in diet between elephant and rhinoceros across seasons that increased towards the dry season, when both diets converged on browse. Admittedly, this trend could also be interpreted as evidence of resource partitioning that enabled these megaherbivores to coexist [16]. Thus, it may not necessarily indicate current competitive displacement, but rather some *ghostly* remnant of past competition [44]. We make no attempt here to distinguish between the consequences of past and present interactions. However, our results also show that rhinoceros diet varied across seasons in different ways, depending on the presence and absence of elephant (see below). This suggests at least tentatively that the trend of increased diet separation may be evidence of current displacement caused by elephant. The separation was characterised by the differential use of shared items (as opposed to the exclusive use of items), which we presume reflects the intensity of competition and the wide and tolerant feeding habits of megaherbivores (that limit the opportunities for exclusive use; [6]). Thus, although we expected the diets to diverge strongly owing to the near-exclusive utilization of grasses by elephant (and avoidance by rhinoceros; [6], [10]), these were only more abundant in elephant diet during summer and were utilised extensively by rhinoceros (up to 63% of the diet in some individuals) throughout. We recorded similar patterns of abundant grass utilisation by rhinoceros in AMC prior to the present study, coinciding with our estimate of their diet where elephant were absent. That is, from August 2001 to April 2002 grass contributed on average 23.8% (SD = 11.5%) of the diet, and up to 47% in some individuals (Landman Unpublished data). The agreement between these findings lends support to the assumption that diet differences between sites are a response to the effects of elephant, rather than sample period. This is despite the fact that black rhinoceros are generally considered to be strict browsers, even in open grasslands: grass contributed<5% of their foraging in 22 published accounts of the diet (e.g. [6], [45]), including the description by Hall-Martin et al. [46] for a site in AMC without elephant. However, our interpretation of these results may be confounded, as previous studies used mostly direct observation or feeding-track techniques, which are vulnerable to underestimating the consumption of grasses (and forbs). Data from Parker et al. [47], showing 15% grass utilisation, should also be treated with caution, as their faecal technique was unusually biased toward the selective retention of grasses. Nevertheless, the evidence of low grass consumption by black rhinoceros demonstrates the importance of our findings and the strength of our comparative approach, despite its geographic limitations. Finally, when elephant reduced their intake of grasses during the dry season, the diets diverged, with rhinoceros utilising more forbs and sharing fewer of the dominant foods (mostly woody shrubs) with elephant. These results are broadly similar to the few studies that evaluated patterns of resource sharing between elephant and other large browsers in relation to changing food availability (e.g. [13]–[15]). However, in most cases, elephant maintained extensive diet separation by utilising different plant species and plant parts. Given the complex spatial and temporal interactions between large herbivores and their food resources, we can only presume that previous studies were unable to detect competition with elephant because food availability was not limiting - a necessary requirement for competition, which we ourselves were unable to demonstrate in this observational study. Nevertheless, the complexity of interactions reveals the merits of a dynamic approach (e.g. determining shifts in resource use in response to the presence of a competitor) to testing competitive interactions [18].

Our second line of evidence comes from our demonstration that rhinoceros diet selectivity can differ in relation to elephant presence. Specifically, we found that rhinoceros in the absence of elephant increased their diet breadth and shifted their diet along the grass-browse continuum (elephant present: 20.8% grass, 79.2% browse; elephant absent: 6.1% grass, 93.9% browse), and in relation to availability. Importantly, rhinoceros switched their preferences for grasses such that these were avoided foods where there were no elephant. We expected the broader diet to include novel items that were either monopolised by elephant in AMC or had disappeared from this site due the impacts of elephant [21], [41]. Instead, we show that the increased breadth was characterised by a change in the abundances of shared items, such that a greater variety of foods comprised the bulk of the diet where elephant were absent. Thus, our study tentatively suggests the role of competition in shaping the food niche of rhinoceros: elephant may have partially excluded rhinoceros from browse resources and regulated their intake of the dominant foods. Although these results cannot be generalised because of a lack of replication across multiple sites, they are broadly consistent with the diet shifts observed between other large herbivores in response to competition (e.g. shifts in diet separation between herbivores [4], [5]; shifts along the grass-browse continuum [48], [49]). Exploration of the mechanism (exploitative and/or interference) of the competitive interaction suggested by our results will be an important area of future research, particularly as both reduced browse availability [22], [41], [42] and agonistic interactions between elephant and rhinoceros have been recorded for AMC [21]. Intriguingly, these interference behaviours were recently confirmed as rhinoceros are the only large herbivores that change their activity patterns in the presence of elephant (as opposed to predators for the other herbivores) in AMC (CJ Tambling Unpublished data). We believe that it is unlikely that the observed shift in rhinoceros diet in relation to elephant could be a consequence of intraspecific competition because Hall-Martin et al. [46] showed that rhinoceros in AMC were able to maintain their expected foraging niche (i.e. limited grass utilisation) despite extensive transformation of habitat at densities *c.* 5 times that of the current study.

The reduced intake of preferred foods and change in diet along the grass-browse continuum has been shown to reduce diet quality in ungulates, with consequences for life-history traits (e.g. body mass and reproduction; [50], [51]). In our study, however, rhinoceros diet quality generally did not vary between sites, despite a significant shift in composition. Instead, the inclusion of grasses (particularly the highly nutritious *C. dactylon*) played an important role in maintaining constant N_f_ levels, while rhinoceros were seemingly able to tolerate the elevated fibre concentrations (fibre content of grass usually exceeds that of browse – [52]) through reduced retention times as hindgut fermenters [53]. The reduced P_f_ levels in AMC are consistent with results for elephant at the same site [54]. While the causal mechanism of this decline remains unclear, we presume that it reflects either a site-effect and/or a consequence of the long-term browsing impacts of elephant [21]. The implication of the latter is that a nutritional decline to rhinoceros will likely arise through reduced phosphorous. Thus, apart from the greater proportion of grass in rhinoceros diet in the presence of elephant, it will be necessary to determine the dietary differences that have contributed toward observed nutritional differences. It is important to recognise, however, that despite the ability of rhinoceros to maintain constant diet quality (and therefore the possible interpretation that elephant facilitate foraging opportunities, rather than compete for food with rhinoceros – e.g. [55]), their lack of specialised grazing adaptations (such as the trunk for elephant – [6], [10]) may increase foraging costs, through reduced harvest- and handling-efficiencies of grasses [53]. In the short-term, we predict that the apparent increase in time spent foraging may be off-set by an enhanced tolerance for low quality food and by seasonally mobilising fat reserves [6], [56]. Although the long-term fitness consequences require exploring, these may be masked by the metapopulation management strategy of black rhinoceros in the Addo Elephant National Park.

In conclusion, our study suggests that competition for food between elephant and other browsers may intensify in fenced areas (created through physical or figurative barriers – [57]) where populations expand and food availability declines. However, in larger, open systems, similar scenarios may arise within shared, preferred habitats. As an example, the conversion of tall riparian woodlands to open habitat along the Chobe River, Botswana, has caused a decline in the abundances of browsing bushbuck *Tragelaphus scriptus ornatus* [43]. Although the mechanism of this decline remains unclear, it is likely that it partly (see [58] for the effects of reduced woody cover) reflects a decline in food availability. Our findings are important for three reasons. First, nearly 90% of South Africa's elephant populations (but not numbers) are currently confined to small enclosed areas similar to AMC [59]. Second, in many cases, browse resources are expected to continue to decline as elephant populations expand in the absence of density-dependent population regulation [10], [60]. Finally, because elephant also play a key role in facilitating access to resources for large herbivores [6], [11], there likely exists a level of elephant utilization that maximises foraging opportunities [61], which need to be quantified and managed.

## Supporting Information

Figure S1 Mean accumulation curves (50 random iterations) of plant species recorded per faecal sample for elephant and rhinoceros.
(TIF)

Figure S2 Non-metric Multidimensional Scaling ordination of principal dietary items identified in the diet of black rhinoceros in the presence (Ep) and absence (Ea) of elephant.
(TIF)

Table S1 Percent contribution (mean ± SD) and preferences of principal dietary items identified in the diet of elephant and black rhinoceros in the Addo Elephant National Park. Symbols+or – show significant preference or avoidance, respectively; dashes indicate that the item was not recorded in the diet; n-PDI, non-principal dietary item.
(TIF)
