# Supplementary figures and images for: Shift in Black Rhinoceros Diet in the Presence of Elephant: Evidence for Competition?
Source: PLoS One. 2013 Jul 17;8(7):e69771. doi: 10.1371/journal.pone.0069771 (PMC3714249; doi:10.1371/journal.pone.0069771)

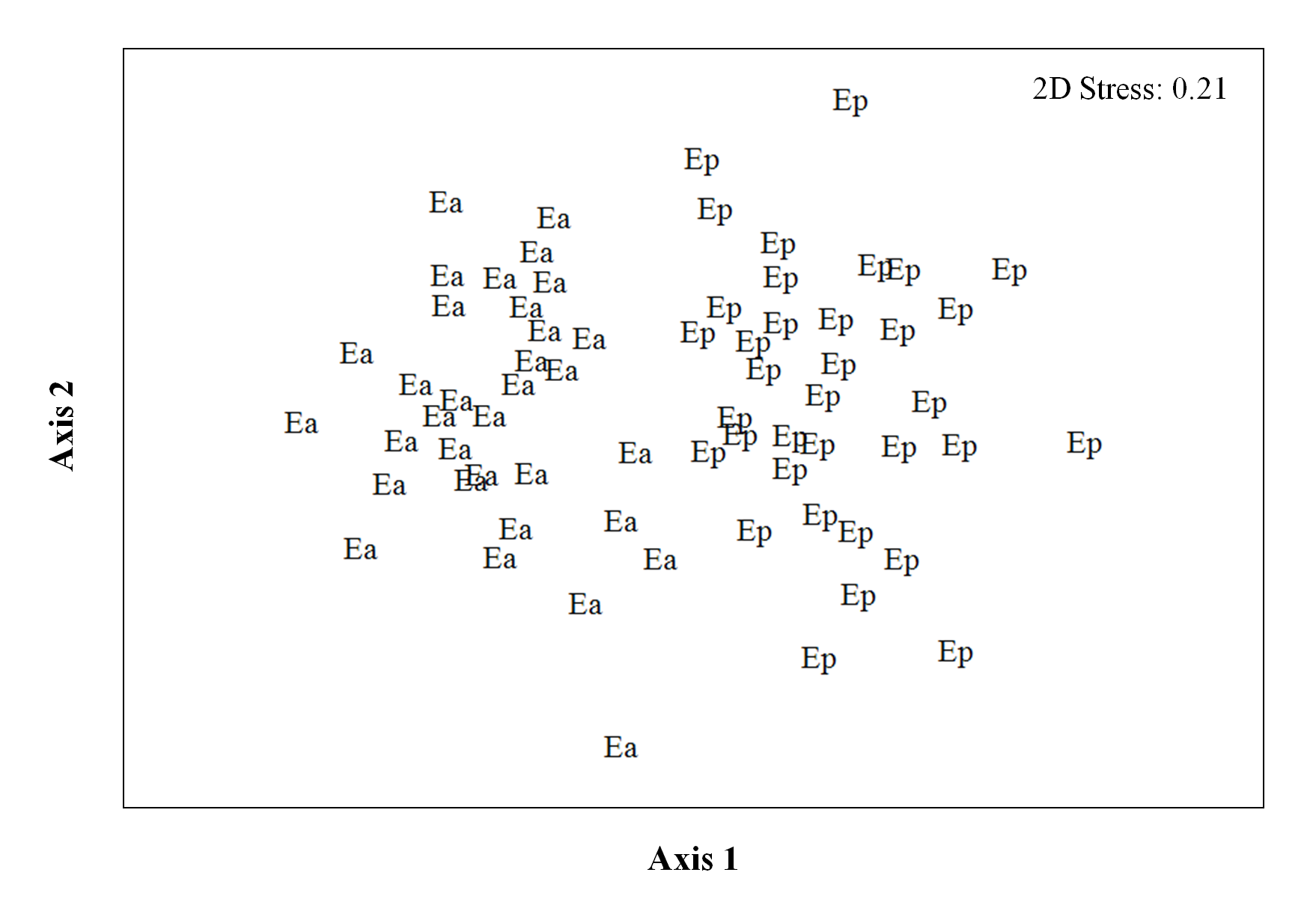

Supplement: Figure S2 — Non-metric Multidimensional Scaling ordination of principal dietary items identified in the diet of black rhinoceros in the presence (Ep) and absence (Ea) of elephant. (TIF) [file pone.0069771.s002.tif]

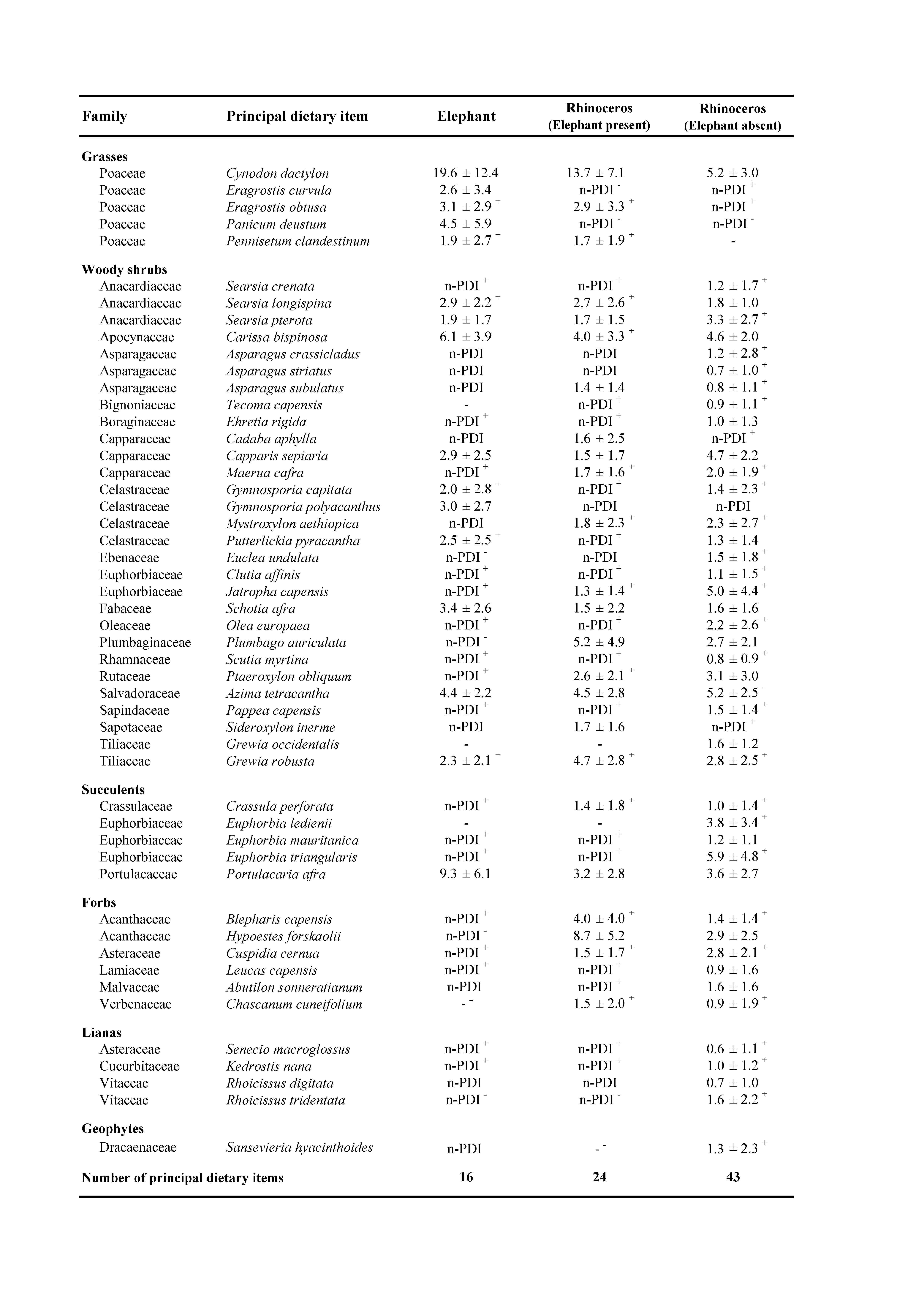

Supplement: Table S1 — Percent contribution (mean ± SD) and preferences of principal dietary items identified in the diet of elephant and black rhinoceros in the Addo Elephant National Park. Symbols+or – show significant preference or avoidance, respectively; dashes indicate that the item was not recorded in the diet; n-PDI, non-principal dietary item. (TIF) [file pone.0069771.s003.tif]
